# Workshop report on the 2^nd^ Joint ENCCA/EuroSARC European bone sarcoma network meeting: integration of clinical trials with tumour biology

**DOI:** 10.1186/2045-3329-4-4

**Published:** 2014-05-01

**Authors:** Jakob K Anninga, Anne-Marie Cleton-Jansen, Bass Hassan, M Fernanda Amary, Daniel Baumhoer, Jean-Yves Blay, Laurence Brugieres, Stefano Ferrari, Heribert Jürgens, Beate Kempf-Bielack, Heinrich Kovar, Ola Myklebost, Michaela Nathrath, Piero Picci, Peter Riegman, Marco W Schilham, Ranin Soliman, Dan P Stark, Sandra Strauss, Matthew Sydes, Patrick Tarpey, David Thomas, Jeremy Whelan, Miriam Wilhelm, Manal Zamzam, Hans Gelderblom, Stefan S Bielack

**Affiliations:** 1 Leiden University Medical Center, Leiden, Netherland; 2 Oxford University, Oxford, UK; 3 Royal National Orthopaedic Hospital, London, UK; 4 University Hospital, Basel, Switzerland; 5 Centre Leon Berard, Lyon, France; 6 Institute Goustave-Roussy, Villejuif, France; 7 Istituto Ortopedico Rizzoli, Bologna, Italy; 8 University Hospital Muenster, Muenster, Germany; 9 Klinikum Stuttgart- Olgahospital, Stuttgart, Germany; 10 Children’s Cancer Research Institute, Vienna, Austria; 11 Norwegian Radium Hospital, Oslo, Norway; 12 TU Munich & Klinikum Kassel, Kassel, Germany; 13 Erasmus Medical Center, Rotterdam, Netherlands; 14 Children’s Cancer Hospital Egypt (57357), Cairo, Egypt; 15 The Leeds Teaching Hospitals, Leeds, UK; 16 University College London Hospitals, London, UK; 17 Medical Research Council Clinical Trials Unit at UCL, London, UK; 18 Sanger Institute, Cambridgeshire, UK; 19 Peter MacCallum Cancer Centre, Melbourne, Austria

**Keywords:** Osteosarcoma, Ewing sarcoma, Bone tumours, Translational research, ENCCA, EuroSARC

## Abstract

This is the report of the 2^nd^ Joint ENCCA/EuroSARC European Bone Sarcoma Network Meeting held in Leiden, The Netherlands, on 26-27 September 2013, bringing together preclinical and clinical investigators on bone sarcoma. The purpose of this workshop was to present the achievements of biological research and clinical trials in bone sarcomas and to stimulate crosstalk.

## Meeting organization

The 2^nd^ Joint ENCCA/EuroSARC European Bone Sarcoma Network Meeting held in Leiden, The Netherlands, on 26-27 September 2013, brought together preclinical and clinical bone sarcoma investigators. The meeting was organized by Stefan Bielack, representing ENCCA (European Network for Cancer research in Children and Adolescents, Work Package 7 (Bone sarcomas)), Bass Hassan, representing EuroSARC (EUROpean clinical trials in rare SARComas within an integrated translational trial network), Anne-Marie Cleton-Jansen representing both and Pauline de Graaf (Leiden, The Netherlands), representing both. The purpose of this workshop was to present the achievements and current status of biological and translational research and clinical trials in bone sarcomas and to stimulate crosstalk. This manuscript is a brief summary of the meeting, which was divided into five sessions.

### Biology/genetics: osteosarcoma

Anne-Marie Cleton-Jansen showed results of genome wide gene expression profiling (Illumina Human-6 v2.0) of osteosarcoma biopsies collected within the EuroBoNet consortium. When comparing profiles from patients who developed metastases within 5 years with those who did not, 50% of the differentially expressed genes were macrophage associated thereby suggesting that macrophages prevent metastases. This beneficial effect of tumour associated macrophages may provide a rationale for the use of macrophage activators such as Liposomal Muramyl-Tripeptide-Phosphatidylethanolamine (L-MTP-PE, Mifamurtide). Furthermore, the Insulin-like Growth Factor 1 receptor (IGF1R) and PI3K/AKT pathways were overactive in osteosarcoma, which in vitro could be inhibited by the dual IR/IGF1R inhibitor Linsitinib (OSI-906) and the Akt-inhibitor MK-2206, respectively.

Fernanda Amary presented the preliminary results of a study on expression of fibroblast growth factor receptor, type 1 (FGFR1) in 288 osteosarcomas. Potential biomarkers for stratification into cohorts which may be candidates for treatment with selective FGFR 1-inhibitors were discussed.

Patrick Tarpey highlighted the role of the International Cancer Genome Consortium (ICGC) in the molecular investigation of bone tumours. Analysis after whole genome, exome, and transcriptome sequencing revealed heterogeneous variations of known and new cancer genes and pathways, including copy number changes, truncating mutations, homozygous deletions, rearrangements and amplifications. Future work will be to establish a dedicated panel of genes for the identification of drivers and pathways, and analysis of the data in the clinical context of subtypes, prognosis and potential targets for treatment.

Ola Myklebost discussed the importance of characterisation of tumour models in terms of tumourigenicity, colony forming ability, proliferation and invasion/migration. The next step, as a prerequisite for personalized treatment strategies, will be deep sequencing to screen for genome wide gene or pathway mutations, or to detect candidate targets. Detected targets can then be evaluated in preclinical models, and, if successful, investigated in second line treatment in patients with refractory disease.

Michaela Nathrath demonstrated that complex genomic alterations in osteosarcoma could be analysed in detail with different genome platforms. Using SNP6.0/Cytoscan arrays, gains, losses and translocations in the Myc-gene resulted in a chromothripsis-like pattern with a poor prognostic significance. Exon sequencing of other non-randomly distributed regions yielded 19 genes with significantly (p = 0.01) different copy number levels, some of which were verified by expression arrays, some of these being potential drug targets.

Daniel Baumhoer presented information on microRNA expression and TP53 alterations in osteosarcoma. In normal tissue, TP53 inhibits the miRNA-17-92 cluster, which regulates cellular processes as cell cycle promotion, apoptosis, senescence and DNA repair. However, in osteosarcoma, the miRNA-17-92 cluster is upregulated which might be influenced by inactivation of TP53. Further, alterations in the first intron of TP53 were detected by whole genome sequencing as a potentially osteosarcoma specific finding.

Piero Picci discussed a revision of histopathologic classifications of bone tumours from the Rizzoli bone tumour bank. Some diagnoses (fibrosarcoma and undifferentiated pleomorphic sarcoma) were less common after review and others (leiomyosarcoma, synovial sarcoma, myoepithelioma) were now more frequent. Also, new primary bone tumour entities were recognized, such as myofibroblastic sarcoma , solitary fibrous tumour , myxofibrosarcoma, and others.

The immunobiology of osteosarcoma was discussed by David Thomas. The role of IL-6, determined by the senescence-associated secretory phenotype, was demonstrated in an irradiated mouse model. IL-6 protects DNA-damaged cells from malignant growth by cell cycle arrest/senescence, whereas IL-6 deficiency results in malignancy. This may be of importance for osteosarcoma, as it has been shown that IL-6 levels can rise after L-MTP-PE infusion.

### Studies & new hypotheses: osteosarcoma

Matthew Sydes presented the first results of the EURAMOS-1 (NCT00134030). Good Response (GR) randomization, assessing pegylated interferon-α2b (ifnα). Of 2.260 registered patients, 1.041 had a known GR and 715 of these were randomized: 358 to MAP (methotrexate, adriamycin, cisplatin), and 357 to MAPifnα (MAP followed by ifnα for 18 months). 23% patients allocated to the MAPifnα arm did not start ifnα (main reason: refusal); 128 patients (55%) completed ifnα, 106 (45%) terminated early (toxicity, progression of disease, refusal, other reasons) and 37 were still on treatment at data freeze (Mar-2013). The main toxicity was haematologic, grade 4 in 7% of the patients, grade 3 in 23%. Data were presented now because the pre-specified number of events had been reported. The hazard ratio (HR) favoured the addition of ifnα, but the confidence intervals (CI) were wide: HR 0.82 (0.61-1.11, 95% CI). Three-year event-free survival (EFS) was 77% for the MAPifnα arm vs. 74% for MAP. The conclusion was that in rare diseases, multinational randomised controlled trials are needed and feasible, and that the (preliminary) estimate of primary outcome favours MAPifnα, although without statistical significance. The CI of the HR includes 1, and the proportion of patients that did not start or complete ifn complicates the interpretation [http://meetinglibrary.asco.org/content/112749-132]. Follow-up will continue on all patients for survival.

A French osteosarcoma trial, randomising between the addition of zoledronic acid (ZA) for 10 months to chemotherapy (OS2006, NCT00470223) was discussed by Laurence Brugieres. The rationale to use ZA was given by animal models. The chemotherapy backbone consists of MTX-IFO/ETO in patients <18 and ADR/DDP/IFO-ADR/DDP in adults >25. Post-operative chemotherapy is adapted to histologic response and the risk group. It is left to the discretion of the centre to use either the MTX or ADR/DDP/IFO-ADR/DDP-based regimen in patients between 18 and 25 years. The primary endpoint of this study is EFS; secondary endpoints are overall survival (OS), histologic response, toxicity and quality of life. Four hundred and seventy patients have to be randomised to make statistically relevant conclusions. Since the start of the study (April 2007), 478 patients have been registered, and 288 have been randomised. So far no significant ZA-related toxicity, except hypocalcaemia, has been reported. Extension of the study to other groups was offered.

An Italian protocol for the treatment of localized osteosarcoma based on P-glycoprotein (PgP) expression (ISG/OS-2, NCT01459484) was presented by Stefano Ferrari. PgP-negative patients are treated with MAP pre-and postoperatively. PgP-positive patients receive MAP preoperatively, and GR patients continue postoperatively with MAP-MTP, whereas poor response (PR) patients are treated with a high-dose IFO-based regimen and MTP. The study started in 2011, and 78 patients have entered the study.

In addition, Ferrari presented the interim results of the EUROpean Bone Over 40 Sarcoma Study (EURO-B.O.S.S.). Here, a chemotherapy backbone of ADR, DDP and IFO - with MTX added to preoperative chemotherapy only in case of PR - is recommended for patients with high-grade bone sarcomas aged 41-65 years. To date, 398 patients with a median age of 52 years have been registered and 344 were deemed evaluable for an interim analysis: 74% had localized disease, the top histological diagnoses were osteosarcoma (primary 49%, secondary 7%), and dedifferentiated chondrosarcoma (13%). Chemotherapy could be given according to the recommendation in most patients and there was only one toxic death. Neutropenia grade 4 was the most frequently reported toxicity. Five-year OS was 55% for the whole group and 65% with surgical remission. OS in primary and secondary osteosarcoma were similar, and poorest in dedifferentiated chondrosarcoma. It was concluded that (complete) surgery is highly important in bone sarcoma in older patients, and that the prognosis with appropriate therapy may not be that different from younger patients. Furthermore, chemotherapy in this age group was feasible, but toxic.

Interim findings from the European Relapsed Osteosarcoma registry (EURELOS), a project endorsed by the European Musculo-Skeletal Oncology Society EMSOS and ENCCA WP7, were presented by Stefan Bielack. To date, 470 patients with a (first) relapse after 1^st^ line treatment had been registered by three collaborating European groups. Patient and relapse characteristics observed in this prospective registry mirrored those of earlier retrospective analyses, but the prospective nature of data collection also allowed hitherto unanswered questions to be addressed, as the contribution of routine surveillance imaging or potential correlations between the use of specific agents and outcomes. It was concluded that international collaboration in relapsed OSA is feasible, but demanding, and that this initiative may help to define standards of care. Participation will be offered to other groups after appropriate protocol amendment.

Bass Hassan discussed the planned EuroSARC MEMOS-study, which will use a Bayesian approach to investigate immunologic biomarkers in metastatic osteosarcoma. Patients with resectable relapsed/metastatic osteosarcoma will be evaluated on their response after 6 weeks L-MTP-PE, and continue for 30 weeks L-MTP-PE after resection of the metastases. Patients deemed unresectable will be randomised between 6 weeks IFO with or without L-MTP-PE, will have a biopsy of one of their lesions, and both groups will continue with IFO plus L-MTP-PE for a total duration of 30 weeks. The primary objective of the study is macrophage infiltration and innate immune activation in the biopsy samples. The secondary objectives are to monitor the safety, tolerability and response rate of L-MTP-PE, in terms of progression-free and overall survival. It is aimed to open the study in 2014.

Improving the cytotoxicity of the innate immune system against osteosarcoma was presented as a promising approach by Marco Schilham. In vitro studies have previously shown that cetuximab can enhance NK-mediated tumour cell killing. Furthermore, M1 and M2 macrophages can both be induced to inhibit growth of osteosarcoma cells in vitro. Activation of the M1-subpopulation with L-MTP-PE + ifnγ induces a soluble factor with anti-tumour activity, which may be an argument in favour of the use of macrophage activating agents as L-MTP-PE in osteosarcoma patients. In addition, IL-10 stimulated M2-macrophages can inhibit growth of osteosarcoma cells in the presence of tumour specific antibodies.

### Interaction: bone sarcomas

Anne-Marie Cleton-Jansen presented the achievements of the EuroBoNeT/ENCCA-WP7 biobank (http://eurobonet.pathobiology.eu/cd/index.php). This web-based virtual biobank has developed standard operating procedures (SOPs) referring to uniform storage and handling of frozen and paraffin tissue samples according to ethical guidelines. In addition, data on cell lines, xenografts, tissue arrays and immunohistochemical (IHC)-reagents for bone tumours are stored. Notably, the material remains at the institute of origin, and only parts of the material are distributed where a request is made to the biobank. Peter Riegman illustrated how samples could be exchanged between partners for translational research. The sample exchange platform is based on a closed TuBaFrost project (http://www.tubafrost.org), and operates within a protected project environment. Samples or pseudonymised data can be requested via a catalogue or a trial locator.

Miriam Wilhelm discussed clinical aspects of osteosarcoma in Teenagers and Young Adults (TYA). Bone sarcomas are typical malignancies of TYAs (3%-5% of all cancers, compared to ≤ 1% in older patients). TYA is also a period with numerous physical, cognitive and behavioural changes which may affect not only the pharmacology of drugs. Several recent studies and meta-analyses observed that survival probabilities for TYA with osteosarcoma lagged behind that of children. Within the TYA cohort, females seem to have better outcomes than males. Age and gender-specific differences in toxicity were also observed. Miriam Wilhelm then shared information from the EURAMOS-1 trial which showed a lack of centralisation and trial participation for the young adult population. These data underline the need for specific care and research for TYA.

Approaches to bone sarcoma research in adolescents and young adults were presented by Dan Stark. The complexity of care is apparent and challenging in this age group. Both international and UK data suggest poorer clinical outcomes of bone sarcomas in TYA than in children or adults, including response to therapy and survival, which is difficult to explain. Factors such as delay in diagnosis, poor trial recruitment, lower adherence to treatment or differences in biology or pharmacology may contribute to this, and have started to become subject of research within Europe and elsewhere. However, more research is still needed. Wider approaches to challenges in European TYA care may include public and patient involvement, evaluating the impact of specialist TYA care, and improving TYA content in professional training curricula.

Manal Zamzam and Ranin Soliman presented the clinical and research activities for bone sarcomas of the Children’s Cancer Hospital Egypt (CCHE). An overview of the paediatric oncology service and auxiliary services, which capture 1200-1400 new patients with paediatric cancers each year, were highlighted. The research department consists of the Research Strategic Business Unit and units for standards of treatment protocols, epidemiology, clinical trials, translational research, biostatistics/informatics, a biorepository, a grant office and an education unit. In addition, CCHE has initiated a fellowship in Paediatric Oncology with Boston Children’s Hospital. From July 2007, data and samples from 166 osteosarcoma patients have been collected. Five-year OS for localized osteosarcoma (n = 100) was 76%, 5-yr EFS 63%.

### Biology/genetics: Ewing sarcoma (ES)

Heinrich Kovar (Vienna, Austria) reviewed current insights into the molecular biology of Ewing sarcoma (ES) and the emerging evidence for the important role of epigenetics. He introduced the concept of the disease arising from a pluripotent mesenchymal stem cell as a consequence of an EWS/FLI1-induced epigenetic imbalance leading to differentiation arrest and unrestricted proliferation. Based on this concept, he postulated that the sarcoma initiating aberration, the chimeric transcription factor, needs to be present at a specific developmental stage during mesenchyme differentiation at which the epigenetic landscape allows EWS/FLI1 to access transforming gene sets. In tumour cells, EWS/FLI1 is found at two distinct genomic locations, at proximal promoters associated mostly with gene activation, and distal (>4 kb) regulatory elements of predominantly repressed genes. A recent GWAS study of 401 ES, which were compared with 4,352 controls, revealed an association of ES with nucleotide polymorphisms on chromosomes 1, 10 and 15. Heinrich Kovar also reported on European collaborative efforts applying integrated genomics, including epigenomic profiling, to identify the mechanism of how these polymorphisms may affect Ewing sarcoma susceptibility.

Piero Picci discussed the role of miR34a in ES. The small inhibiting RNA miR-34a is a transcriptional target of TP53 and induces apoptosis and cell death by silencing, amongst others, CDKs, BCL2, nMyc and SIRT1. Using qRT-PCR analysis it was shown that high miR-34a in ES predicted survival and proved to be a significant prognostic marker in multivariate analysis. qRT-PCR proved to be more robust as a marker for survival than In-Situ-Hybridization, and may be useful for patient stratification at diagnosis in international studies.

David Thomas discussed the International Sarcoma Kindred Study (ISKS).The ISKS is a unique biological, epidemiological and clinical study on sarcoma patients and their families, and a resource created to investigate the heritable aspects of sarcoma (http://www.australiansarcomagroup.org/sarcomakindredstudy). The study started in Australia, but has partners in Canada, USA, France and the UK. Of 831 registered patients, more than 100 have a malignant bone tumour. The study is now moving from single gene studies to complex, next-generation sequencing studies.

### Studies & new hypotheses: Ewing sarcoma

Heribert Juergens gave an overview of the evolution of ES trials in Europe. In total 4,659 patients have enrolled in CESS 81, CESS 86, EICESS 92 and EE99. Three-year EFS improved from 56% to 71%. Systemic treatment moved from 4 (VACA) to 6 drugs (VAC/IA (E)). Adding IFO and ETO in addition to interval compression seems beneficial for non-metastatic ES, but in patients with metastatic disease or poor response to preoperative chemotherapy, the best treatment needs to be defined. The addition of radiotherapy to surgery seems to have improved local control, irrespective of the response to chemotherapy. The first results of the EE99 standard (R1) arm randomisation between cyclophosphamide and IFO have shown no significant difference in 3y-EFS or -OS. The R2-arm still is recruiting patients because the target to answer the randomisation question has not yet been met. It was concluded that intensive treatment in ES is essential and combined local treatment is preferable to improve outcome.

Heribert Juergens went on to discuss EWING-2008 (NCT00987636), a study in localised and disseminated ES. Randomisation in the R1-arm is between add-on treatment with zoledronic acid or no add-on treatment after VAC (females) or VAI (males) maintenance. The R2-arm is continued from EE99, and a very high-risk arm (R3) examines the potential benefit of HD-chemotherapy with stem cell reinfusion added to eight cycles of maintenance chemotherapy. The trial started in 2009, and 313 patients have been included to date. Other approaches for future trials in collaboration with the EORTC or INFORM (Individualized therapy for relapsed malignancies in childhood adolescents and young adults) were briefly presented.

Jeremy Whelan and Sandra Strauss presented the activity of the EURO-EWING consortium (EEC). The EEC is an FP7 funded coalition of European study groups and Sarcoma Patients Euronet (SPAEN), dedicated to improve survival from ES by an integrated programme of investigator-driven trials that are underpinned by complementary translational research. The activities of the EEC include a first-line randomised study comparing VIDE versus an interval- compressed VDC/IE approach to define the standard of care for neo-adjuvant chemotherapy; secondly, a multi-arm randomised trial of 4 second-line chemotherapy regimens in recurrent ES, that serves as a platform for testing new agents; thirdly, to introduce public and patient involvement in the work of EEC.

Bass Hassan presented a planned EORTC-EuroSARC phase-II trial of the dual tyrosine kinase inhibitor for IGF1R and insulin receptor (IR), linsitinib, in patients with relapsed or refractory ES (LINES-study). To develop an informative trial, biomarkers will be validated using exome sequencing and functional imaging of the effect of linsitinib. This validation is expected to lead to a better understanding of the mechanism of action of this dual IGF1R/IR inhibitor.

## Conclusions

The meeting provided a thorough update of biological and clinical studies in both osteosarcoma and ES. Recent clinical trials have not demonstrated impressive improvements in survival, but large collaborations are clearly feasible and desirable, both clinically as well as scientifically. Preclinical studies have provided a wealth of new data, requiring renewed bioinformatic analyses. Continued research is expected to identify new pathways and mechanisms of tumourigenesis, which may then be useful for the identification of biomarkers and the development of targeted therapies. However, the complexity of molecular-genetic events in osteosarcoma and ES has so far impeded the rapid identification of new treatment targets. Furthermore, translation of pre-clinical models into clinical practice is anything but simple, and innovative methods of testing potential drug effects will be needed. Also, limited interest in developing new agents for these rare cancers on the part of industry remains a challenge. Nevertheless, relatively small trials of molecular targeted therapies in well defined cohorts, validated by reliable biomarkers, may be a way to open new doors in the treatment of these tumours.

## Abbreviations

ADR: Adriamycin (doxorubicin); CESS: Cooperative Ewing Sarcoma Study; CCHE: Children’s Cancer Hospital Egypt; CI: Confidence interval; DDP: Cisplatin; EE99: Euro-EWING-99 study; EEC: EURO-EWING consortium; EFS: event free survival; ENCCA, European Network for Cancer research in Children and Adolescents; EICESS: European Intergroup Cooperative Ewing Sarcoma Study; EORTC: European Organisation for Research and Treatment of Cancer; ES: Ewing sarcoma; ETO: Etoposide; EURAMOS: European and American Osteosarcoma Study; EURELOS: European Relapsed Osteosarcoma registry; EuroBoNeT: European Network to Promote Research into Uncommon Cancers in Adults and Children: Pathology, Biology and Genetics of Bone Tumours.; EURO-B.O.S.S: EUROpean Bone Over 40 Sarcoma Study; EuroSARC: (EUROpean clinical trials in rare SARComas within an integrated translational trial network); EWS/Fli -1: Chimeric and oncogenic transcription factor in Ewing sarcoma as result of the translocation between the chromosomes 11 (Fli-1) and 22 (EWS); FP7: European Union’s Seventh Framework Programme for Research and Technological Development; GR: Good histologic response to induction chemotherapy; GWAS: Genome Wide Association Study; HR: Hazard ratio; ifnα: (pegylated) interferon-α2b; ifnγ: interferon gamma; IFO: Ifosfamide; IGF1R: Insulin-like growth factor 1 receptor; IHC: Immunohistochemistry; INFORM: Individualized therapy for relapsed malignancies in childhood adolescents and young adults; IR: Insulin receptor; ISKS: International Sarcoma Kindred Study; FGFR1: Fibroblast growth factor receptor, type 1; L-MTP-PE: Liposomal Muramyl-Tripeptide-Phosphatidylethanolamine, Mifamurtide; MAP: Methotrexate Adriamycin, Cisplatin; MTX: Methotrexate; OS: Overall survival; PgP: P-glycoprotein; PR: Poor histologic response to induction chemotherapy; qRT-PCR: quantitative Real-Time polymerase chain reaction; SOP: Standard operating procedure; SPAEN: Sarcoma Patients Euronet; TYA: Teenagers and Young Adults; VAC: Vincristine, actinomycin, cyclophosphamide; VACA: Vincristine, actinomycin, cyclophosphamide, adriamycin; VAC/IA (E): Vincristine, actinomycin, cyclophosphamide / Ifosfamide, adriamycin (etoposide); VAI: Vincristine, actinomycin, ifosfamide; VDC/IE: Vincristine, adriamycin, cyclophosphamide/ifosfamide, etoposide; VIDE: Vincristine, ifosfamide, adriamycin, etoposide; ZA: Zoledronic acid.

## Competing interests

Consultant/Advisory Board/ Scientific support Speaker’s fees. Stefan Bielack Bayer, Merck & Co, Takeda-Millennium/IDM. Stefano Ferrari: Takeda, MSK, Amgen; Pharmarmar, Morphotek, Amgen, Molmed Heribert Jürgens: Takeda-Medac.

## Authors' contributions

JKA participated in the conception and design of the manuscript, collated the data from all coauthors, led the drafting of the manuscript, and revised the article critically. ACJ participated in the conception and design of the manuscript, provided information regarding her group’s research for inclusion into the manuscript and revised the article critically. BH participated in the conception and design of the manuscript and revised the article critically. MFA provided information regarding her group’s research for inclusion into the manuscript and revised the article critically. DB provided information regarding his group’s research for inclusion into the manuscript and revised the article critically. JYB participated in the conception and design of the manuscript and revised the article critically. LB provided information regarding her group’s research for inclusion into the manuscript and revised the article critically. SF provided information regarding his group’s research for inclusion into the manuscript and revised the article critically. HJ provided information regarding his group’s research for inclusion into the manuscript and revised the article critically. BKB participated in the drafting of the manuscript and revised the article critically. HK provided information regarding his group’s research for inclusion into the manuscript and revised the article critically. OM provided information regarding her/his group’s research for inclusion into the manuscript and revised the article critically. MN provided information regarding her group’s research for inclusion into the manuscript and revised the article critically. PP provided information regarding his group’s research for inclusion into the manuscript and revised the article critically. PR provided information regarding his group’s research for inclusion into the manuscript and revised the article critically. MWS provided information regarding his group’s research for inclusion into the manuscript and revised the article critically. RS provided information regarding her group’s research for inclusion into the manuscript and revised the article critically. DPS provided information regarding his group’s research for inclusion into the manuscript and revised the article critically. SS provided information regarding her group’s research for inclusion into the manuscript and revised the article critically. MS provided information regarding his group’s research for inclusion into the manuscript and revised the article critically. PT provided information regarding his group’s research for inclusion into the manuscript and revised the article critically. DT provided information regarding his group’s research for inclusion into the manuscript and revised the article critically. JW provided information regarding his group’s research for inclusion into the manuscript and revised the article critically. MW provided information regarding her group’s research for inclusion into the manuscript and revised the article critically. MZ provided information regarding her group’s research for inclusion into the manuscript and revised the article critically. HG participated in the conception and design of the manuscript, in the drafting of the manuscript and in coordinating this process, and revised the article critically. SSB participated in the conception and design of the manuscript, provided information regarding his group’s research for inclusion into the manuscript, participated in the drafting of the manuscript and in coordinating this process, and revised the article critically. All authors read and approved the final manuscript.

